# Vertical collision risk of Red-crowned Cranes at coastal onshore wind farms in eastern China

**DOI:** 10.7717/peerj.21762

**Published:** 2026-10-01

**Authors:** Kai Dong, Xinyi Hu, Changhu Lu

**Affiliations:** College of Life Sciences, Nanjing Forestry University, Nanjing, Jiangsu, China

**Keywords:** Wind energy, Collision risk, *Grus japonensis*, Coastal wetlands, Satellite tracking

## Abstract

**Background:**

With the continued expansion of coastal wind farms toward wetland edges, understanding the mechanisms that drive collision risk for large waterbirds around wind turbines is becoming increasingly important.

**Methods:**

Based on satellite tracking data from seven Red-crowned Cranes (*Grus japonensis*) in the coastal area of Yancheng, Jiangsu Province, and the geometric parameters of the wind turbine rotor-swept zone (RSZ), we developed a coupling framework linking crane flight heights with the vertical position of the RSZ. We used this framework to assess potential collision risk during the non-migratory period.

**Results:**

Crane flight height showed clear vertical overlap with the RSZ, but potential collision risk was not evenly distributed throughout the RSZ. Instead, risk was concentrated in several adjacent low to medium altitude bands. The 30–35 m band contributed the most, followed by the 35–40 m and 40–45 m bands. Simulations showed that increasing turbine hub-height could substantially reduce collision risk by shifting the RSZ upward as a whole. A 20 m increase in hub-height reduced overall risk by 48.9%.

**Discussion:**

Based on empirical flight height data, this study revealed the heterogeneous vertical distribution of potential collision risk for Red-crowned Cranes and the mechanisms underlying its formation. It also identified key risk height bands, providing a more mechanistic basis for turbine-design optimization, risk mitigation, and coordination between coastal wind power development and waterbird conservation.

## Introduction

As an important component of clean energy, wind power has expanded rapidly worldwide in recent years (Katzner et al., 2025), increasingly overlapping with coastal regions of high ecological value (Rehbein et al., 2020; Zhao et al., 2024). Although wind power development is widely expected to contribute to the energy transition and climate change mitigation, wind power facilities may also have negative impacts on birds through collisions, migration barriers, and habitat changes (Marques et al., 2014). Therefore, in the context of sustainable wind power development, accurately assessing its potential ecological impacts on birds is essential for balancing clean energy expansion with avian conservation.

Among the various impacts of wind power on birds, collision is one of the most direct and widely recognized ecological risks (Gémard et al., 2025; Roy et al., 2025). Recent studies indicate that collision risk is jointly influenced by birds’ spatial use and flight behavior, species-specific characteristics, habitat context, and turbine characteristics and spatial arrangement (Estellés-Domingo & López-López, 2025). Spatial overlap between bird movements and turbine risk zones is therefore a prerequisite for collision exposure. Therefore, a large number of studies have evaluated the potential conflicts between wind turbines and birds from the horizontal spatial dimension to identify high-risk areas and provide a basis for the macroscopic siting of wind farms (Bose et al., 2020; Balotari-Chiebao et al., 2023; Zhao et al., 2024). However, horizontal spatial studies mainly depict the proximity of birds and turbines in geographical space, and it is difficult to determine whether birds enter the vertical risk space represented by the rotor-swept zone (RSZ). Therefore, vertical collision risk assessment based on flight height and RSZ range has gradually attracted attention.

Some studies have begun to combine bird flight height with the RSZ range to determine whether birds enter the height range of wind turbine blades and to assess the overall overlap between bird flight and the RSZ (Johnston et al., 2014; Cleasby et al., 2015). At the same time, it has been shown that the structural parameters of wind turbines also affect the position and range of the RSZ, thereby influencing the mortality and collision risk of birds and bats (Barclay, Baerwald & Gruver, 2007). During wind farm renovation and upgrading, changes in the size and number of wind turbines may also alter wildlife mortality risk (Loss, Will & Marra, 2013; Huso et al., 2021). Recent studies have further decomposed turbine size into specific parameters, such as the minimum blade height above ground, rotor diameter, and rated power, and found that these parameters have species-specific effects on the mortality of different bird and bat species (Garvin, Simonis & Taylor, 2024). Simulation studies have shown that increasing the minimum blade height, changing rotor diameter, or changing turbine capacity may alter the collision risk of different species (Schaub et al., 2024). Therefore, the RSZ is not a homogeneous vertical space; its position and range are jointly determined by geometric parameters such as hub-height, rotor radius, and minimum blade height above ground (Masden & Cook, 2016; Garvin, Simonis & Taylor, 2024). Although existing vertical risk assessments have focused on the overall overlap between bird flight height and the RSZ, they rarely combine species-specific flight height use with the vertical geometric structure of the RSZ, making it difficult to reveal the height distribution of potential collision risk within the RSZ, key risk height bands, and the mitigation effects of turbine structural adjustments.

To fill this gap, this study focused on the local population of the Red-crowned Crane in the coastal area of Yancheng, Jiangsu Province. It combined satellite tracking based flight height data of Red-crowned Cranes with the vertical geometric structure of wind turbines to assess the potential vertical collision risk of large waterbirds in coastal onshore wind power scenarios. The Red-crowned Crane is an important flagship waterbird in the eastern coastal wetlands of China, and the coastal area of Yancheng is an important wintering and non-breeding area for this species (Xu et al., 2019). In recent years, with the continuous implementation of rescue and rewilding efforts, some released individuals have formed a small local population that stays year round and breeds continuously in the coastal area of Yancheng (Wu et al., 2024). The coastal area and surrounding regions of Yancheng are also areas with relatively concentrated onshore wind power development in eastern China, and there is potential overlap between the daily activity space of Red-crowned Cranes and surrounding wind power facilities.

Based on satellite tracking data from seven Red-crowned Cranes and the geometric parameters of wind turbines, this study addressed three specific questions: (1) whether the flight height of Red-crowned Cranes overlaps vertically with the RSZ; (2) whether potential collision risk is highly heterogeneous within the RSZ and concentrated in specific height bands; (3) whether raising the hub-height and shifting the RSZ upward can reduce potential vertical collision risk to Red-crowned Cranes. By combining species-specific flight height use with the vertical structure of wind turbines, this study aims to reveal the height dependence of potential collision risk in Red-crowned Cranes and provide a case reference for vertical risk assessments of large waterbirds in relation to onshore wind power facilities.

## Materials & Methods

### Study area

This study was conducted in the coastal wetland complex of Yancheng, Jiangsu Province, eastern China. This area consists of various habitats such as intertidal mudflats, reed marshes, and farmlands, forming a typical mosaic of coastal wetlands. Yancheng coastal area is located at a key position on the East Asian–Australasian Flyway (EAAF), and is an important wintering and migration stopover site for the globally threatened species, the Red-crowned Crane (Li et al., 1999; Qiu et al., 2024). In recent years, wind power development has gradually expanded into intertidal zones and wetland edges, bringing wind turbines closer to areas used by Red-crowned Cranes at the local scale and thereby increasing the potential for spatial overlap (Fig. 1).

**Figure 1:**
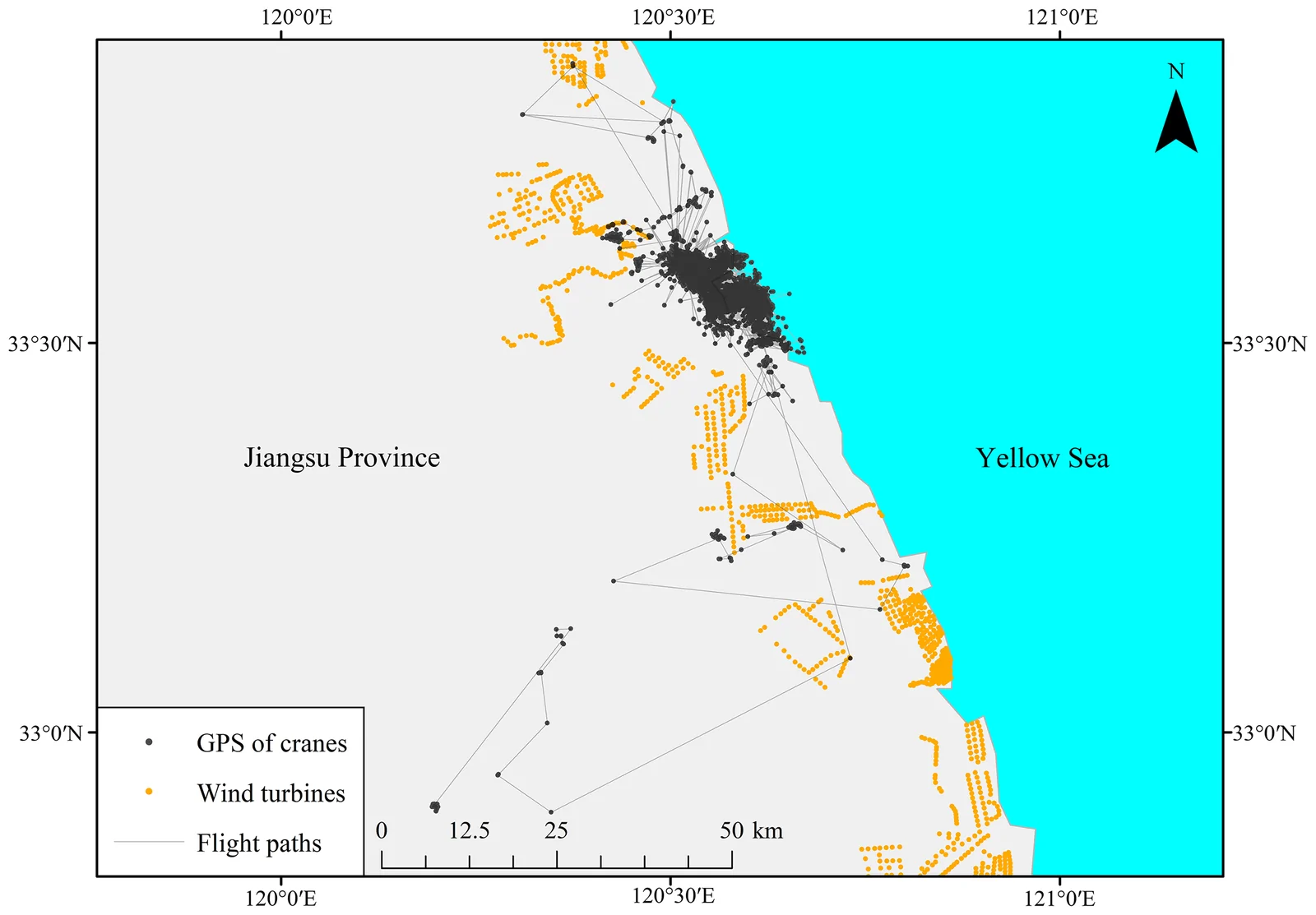
Satellite tracking records of Red-crowned Cranes and the distribution of onshore wind turbines in the Yancheng coastal area. Filtered satellite-tracking records of seven Red-crowned Cranes and the spatial distribution of onshore wind turbines in the Yancheng coastal area, Jiangsu Province, eastern China. Base map imagery was obtained from Tianditu (https://www.tianditu.gov.cn/). Wind turbine locations were derived from OpenStreetMap (https://www.openstreetmap.org/). Red-crowned Crane GPS locations and flight paths were generated from the authors’ satellite-tracking dataset.

### Satellite tracking data

Satellite tracking data for Red-crowned Cranes were obtained from a long-term rescue, rehabilitation, and post-release monitoring program conducted in the Jiangsu Province Yancheng National Nature Reserve for Rare Birds. The seven Red-crowned Cranes included in this study were rescued and released individuals monitored by the reserve. Satellite transmitters (HQLG4037S, 37–44 g; Hunan Global Messenger Technology Co., Ltd., Changsha, China) were attached as part of the monitoring program rather than for the purposes of the present study. The weight of all transmitters was less than 3% of the body mass of each individual, meeting the ethical standards for wildlife telemetry research (Lameris & Kleyheeg, 2017).

Positioning data were transmitted from each transmitter to a remote data server through the Global System for Mobile Communications (GSM) cellular network, with a sampling interval of 1 h. The monitoring period covered November 2022 to October 2025, and each positioning record contained the timestamp, geographic coordinates, positioning elevation, instantaneous flight speed, and other movement information. The accuracy of the recorded flight-height data was approximately 5 m.

The present study did not involve any new fieldwork, animal capture, handling, tagging, specimen collection, or additional transmitter deployment. All analyses were based solely on existing tracking data generated by the long-term rescue-and-release monitoring program. The original rescue, release, and satellite-tracking procedures were reviewed and approved by the Ethics Committee of Nanjing Forestry University. All procedures complied with the institutional animal welfare requirements of Nanjing Forestry University, the Wildlife Protection Law of the People’s Republic of China, and the Guidelines to the Use of Wild Birds in Research (Fair, Paul & Jones, 2010). The principles of Replacement, Reduction, and Refinement (3Rs) were addressed by reusing existing monitoring data and avoiding additional animal capture or handling for the purposes of this study.

To ensure data quality, the original satellite-tracking data underwent systematic preprocessing: (1) records with missing time information or spatial positioning errors were removed; (2) abnormal points with flight speeds exceeding the known flight capabilities of Red-crowned Cranes were excluded; and (3) recorded altitudes were corrected using a digital elevation model (DEM) to calculate flight height above ground level. After filtering, 69,173 flight-height records were retained for the vertical collision risk analysis (Data S1).

### Wind turbine data

Based on the activity range defined by the satellite tracking data of Red-crowned Cranes, this study included only the onshore wind turbines that overlapped with the cranes’ activity areas in the analysis. The spatial position of the turbines was obtained from the OpenStreetMap (OSM) database (OpenStreetMap contributors, 2025) and extracted using the Overpass API through the Overpass Turbo tool (https://overpass-turbo.eu) (Raifer, 2025). To enhance the completeness and spatial accuracy of the data, we manually verified and corrected the turbine positions based on high resolution satellite images, and supplemented the digitalization of the missing turbines. The vertical range of the rotor sweep zone was determined based on the turbine structure parameters provided by the wind farm operator and the relevant information from public technical documents.

### Data analysis

To quantify the potential vertical collision risk caused by the overlap between Red-crowned Crane flight height and the wind turbine RSZ in the vertical dimension, we developed a height coupling analysis framework. Based on the overall statistics of turbine structural parameters in the study area, the vertical range of the rotor-swept zone was defined as 21.5–155.5 m, corresponding to a hub-height of *H* = 88.5 m and a rotor radius of *r* = 67.0 m. These parameters represent the typical vertical geometric characteristics of wind turbines in the study area.

The probability density function of Red-crowned Crane flight height, *p*(*h*), was estimated from quality controlled flight height data using kernel density estimation (KDE) (Silverman, 1986):

\begin{eqnarray*}p(h)= \frac{1}{n\sigma } \sum _{i=1}^{n}K( \frac{h-{h}_{i}}{\sigma } ) \end{eqnarray*}

where *n* is the number of flight-height observations, *hi* is the *i*-th observed flight height, and *h* is the height variable used to calculate the probability density of flight height.

To describe the geometric exposure of the wind turbine at different heights, a height dependent swept chord length function, *L*(*h*), was introduced. This function is defined based on the geometric structure of the rotor disc and is used to represent the swept chord length of the rotor at height *h*.

\begin{eqnarray*}L(h)=\begin{array}{@{}c@{}} \displaystyle \frac{2\sqrt{{r}^{2}-(h-H)^{2}}}{2r} , H-r\leq h\leq H+r. \end{array} \end{eqnarray*}

This function reflects the relative geometric hazard of the turbine rotor at different heights.

Based on the two functions above, the relative vertical collision risk between birds and wind turbines was quantified using the risk index *R*. This index was defined as the integrated coupling of the probability distribution of flight height use and the geometric hazard function of the wind turbine over the height range of the RSZ:

\begin{eqnarray*}R=\int \nolimits \nolimits _{H-r}^{H+r}p(h)L(h)dh. \end{eqnarray*}

*R* represents the relative coupling strength between Red-crowned Crane flight height and the vertical risk of wind turbines (Johnston et al., 2014). It was used to describe relative potential collision risk under different scenarios, rather than absolute collision probability.

To identify the key risk height bands, the RSZ was divided into five m height intervals, and the KDE-based relative flight height use, the mean relative rotor swept chord length, and the relative risk contribution within each five m height band were calculated and ranked accordingly.

In the simulation, it was assumed that increasing hub-height would shift the RSZ upward as a whole. The risk index under each hub-height increase scenario, *R*_Δ*H*_, was recalculated, and the relative risk reduction was calculated as:

\begin{eqnarray*}{\mathrm{Reduction}}_{\Delta H}= \frac{{R}_{0}-{R}_{\Delta H}}{{R}_{0}} \times 100\% \end{eqnarray*}

where *R*_0_ represents the risk index under the baseline hub-height condition, *R*_Δ*H*_ represents the risk index recalculated after increasing hub-height by Δ*H*, and *Reduction*_Δ*H*_ represents the proportion of risk reduction relative to the baseline scenario.

All statistical analyses were conducted in R 4.5.2. Kernel density estimation was performed using the package ‘stats’, and spatial data processing and visualization were carried out using the package ‘sf’ and ‘ggplot2’, respectively (R Core Team, 2025).

## Results

### Height-dependent collision risk

When the flight heights of Red-crowned Cranes were combined with the vertical geometry of the RSZ, collision risk showed a pronounced height-dependent pattern (Fig. 2). The flight heights of Red-crowned Cranes were concentrated at relatively low altitudes (Fig. 2A), while the geometric exposure of the RSZ in the vertical dimension changed with height and reached a maximum near hub height (Fig. 2B). Because of this coupling, a pronounced risk peak emerged in the lower part of the rotor-swept zone (Fig. 2C).

**Figure 2:**
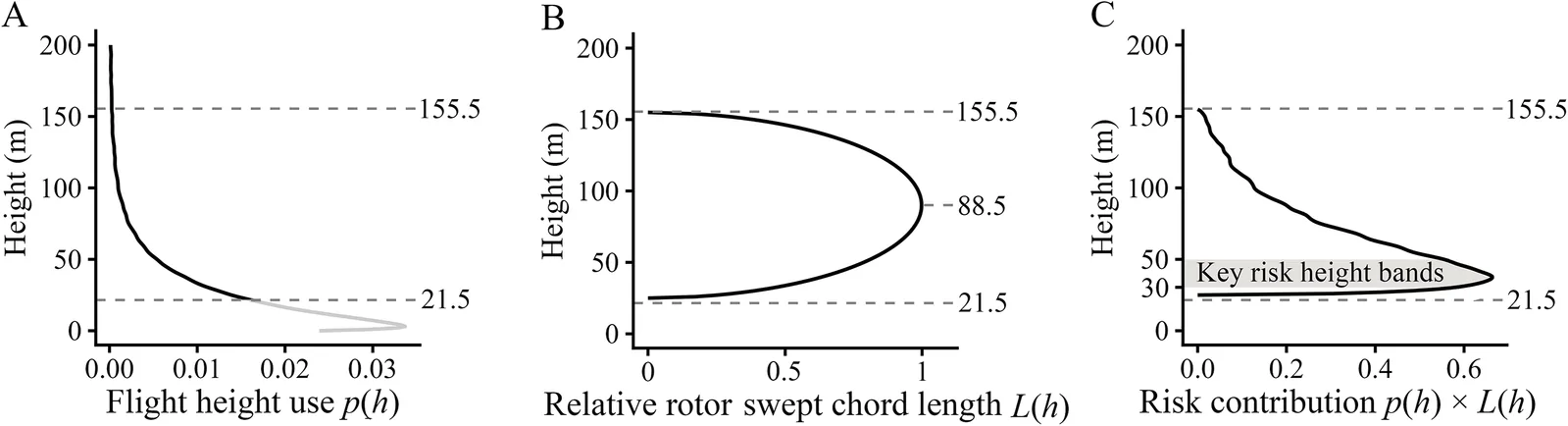
Height dependent collision risk resulting from the vertical coupling between crane flight height use and turbine rotor geometry. (A) Kernel density distribution of Red-crowned Crane flight-height use, *p*(*h*). (B) Relative rotor-swept chord length, *L*(*h*), representing height-dependent geometric exposure within the rotor-swept zone. (C) Relative risk contribution calculated as *p*(*h*) ×*L*(*h*). Dashed horizontal lines indicate the lower and upper limits of the current rotor-swept zone (21.5 and 155.5 m), and the hub height is 88.5 m. The shaded band indicates the key risk height bands.

Therefore, collision risk is mainly concentrated within a limited vertical portion of the RSZ. The ranking of the contributions of different height bands to overall risk showed that the greatest contributions came from several adjacent low to medium height bands, with the 30–35 m band contributing the most, followed by the 35–40 m and 40–45 m bands (Table 1). All height bands across the RSZ are provided in Table S1.

**Table 1:** Height bands ranked by their contribution to relative potential collision risk within the rotor-swept zone.

Rank	Height band (m)	Relative flight height use *p*(*h*)	Mean rotor-swept chord length *L*(*h*)	Risk contribution *p*(*h*) ×*L*(*h*)
1	30–35	12.05	0.55	6.63
2	35–40	10.03	0.65	6.52
3	40–45	8.29	0.73	6.05
4	25–30	13.61	0.41	5.58
5	45–50	6.87	0.79	5.43

### Effects of turbine-design on collision risk

The simulation indicated that potential collision risk decreased continuously with increasing turbine hub-height (Fig. 3; Table 2).

**Figure 3:**
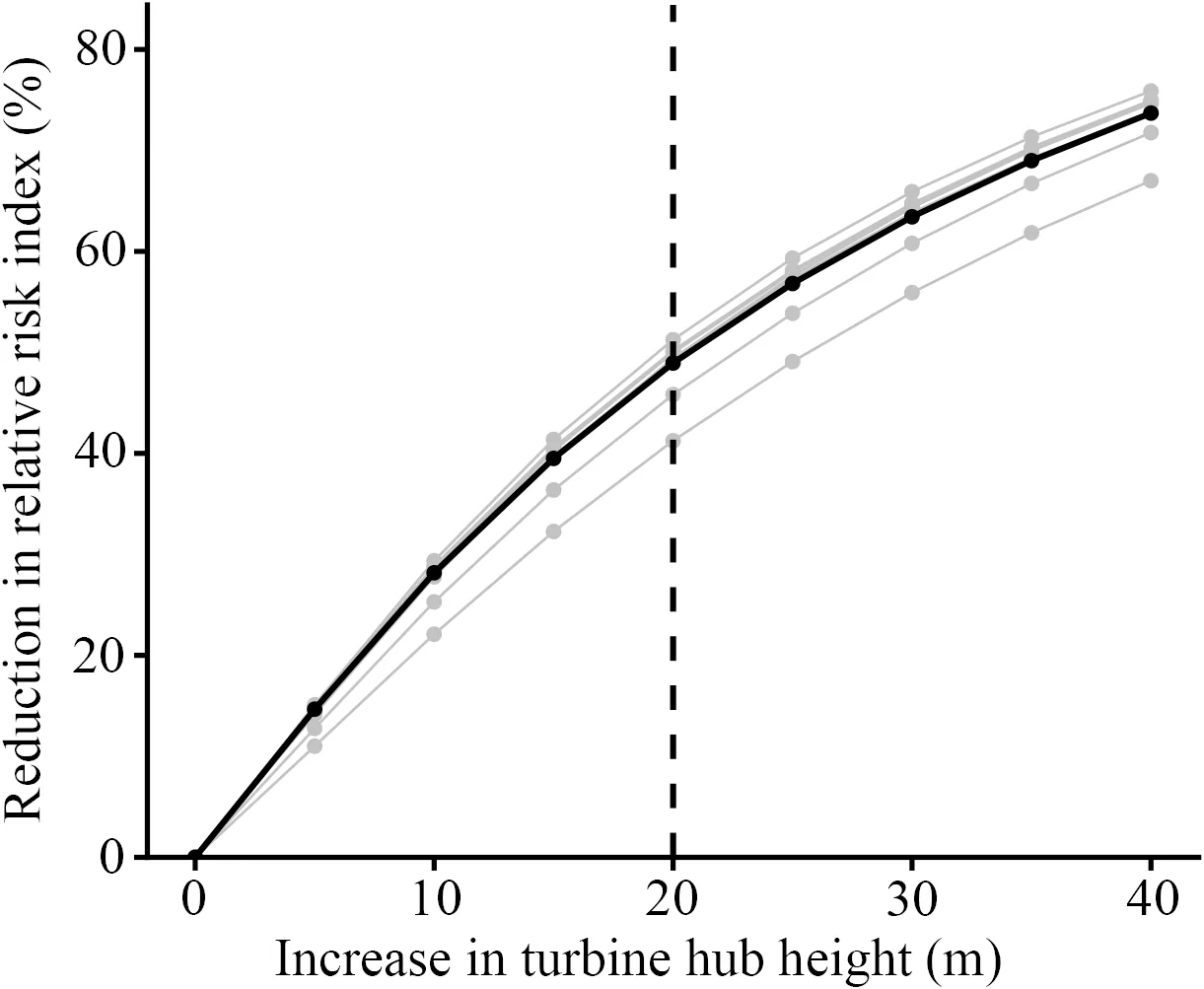
Simulated reduction in relative risk index under turbine hub-height increase scenarios. The black line indicates the mean reduction, grey lines indicate individual-specific reductions, and the dashed vertical line marks the +20 m hub-height scenario.

**Table 2:** Simulated reduction in collision risk under turbine hub-height increase scenarios.

Hub-height increase (m)	Mean reduction (%)	Marginal gain (% per m)
0	0	NA
5	14.7	2.93
10	28.2	2.70
15	39.5	2.27
20	48.9	1.89
25	56.8	1.58
30	63.4	1.32
35	69.0	1.11
40	73.7	0.94

A 20 m increase in hub-height reduced the relative risk index by 48.9%, after which additional reductions became progressively smaller, indicating diminishing returns (Table 2).

This reduction occurred because raising the hub-height shifted the RSZ upward while rotor radius was held constant (Fig. 4), thereby reducing overlap between the RSZ and the low-altitude flight heights most frequently used by Red-crowned Cranes.

**Figure 4:**
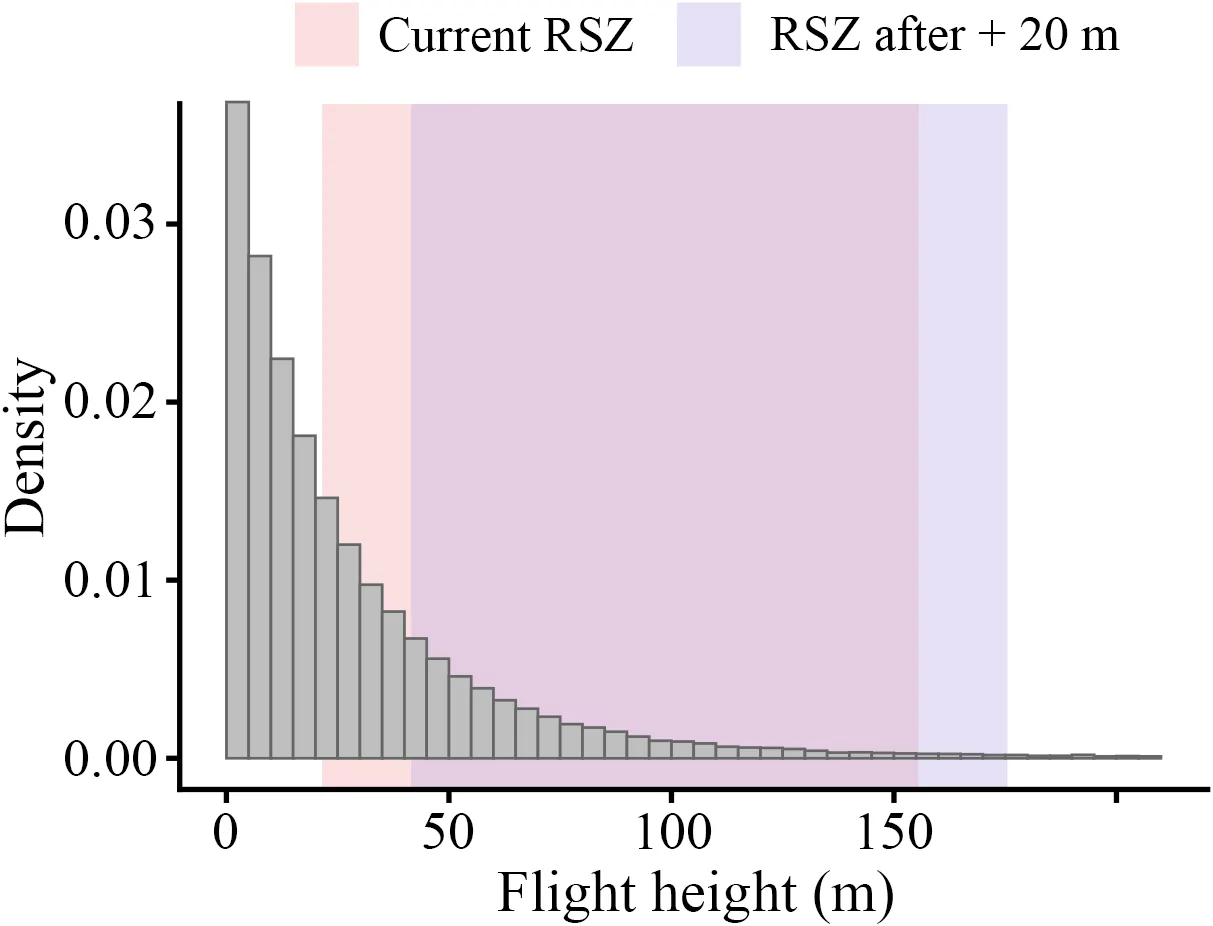
Flight-height distribution of Red-crowned Cranes and simulated upward shift of the rotor-swept zone after a 20 m increase in turbine hub height. The shaded bands indicate the current RSZ (21.5–155.5 m) and the RSZ after the +20 m hub-height scenario (41.5–175.5 m), with rotor radius held constant.

## Discussion

### Vertical heterogeneity of collision risk

This study shows that the potential collision risk between Red-crowned Cranes and wind turbines is mainly concentrated in the lower part of the RSZ, resulting from the overlap between the upper range of the crane flight heights and the lower part of the RSZ. However, the location of high-risk height bands may differ among bird species. Studies of seabirds have also found that the collision risk is often concentrated in the lower part of the RSZ (Johnston et al., 2014). In contrast, groups such as raptors, which more commonly perform high altitude hovering, gliding, or updraft related activities, may use higher flight heights more frequently (Péron et al., 2017; Rolek et al., 2022), indicating that flight height is a key variable in determining the difference in collision risk (Katzner et al., 2012; Furness, Wade & Masden, 2013). At the same time, the position of the key height band is determined not only by species-specific flight characteristics, but also by turbine structure. Turbine structural parameters can alter the vertical distribution of risk, increasing rotor diameter may increase bird collision risk, whereas increasing ground clearance helps reduce it (Škrábal et al., 2025). Hub-height determines the vertical position of the RSZ, while rotor diameter affects its vertical extent and geometric structure at different heights (Garvin, Simonis & Taylor, 2024). This suggests that the key to assessing collision risk at a local scale is not simply determining whether birds enter the RSZ, but identifying the key height bands that actually drive risk under different species and wind turbine structure combinations. Accordingly, the key risk height bands identified in this study should be interpreted as specific to Red-crowned Cranes during the non-migratory period. Although the analytical framework can be applied to other birds, the specific risk height bands should not be directly extrapolated, because flight-height use may vary among species and across seasons, activity types, and breeding status. Species-specific assessments therefore remain necessary.

### Behavioral mechanism

The key risk height bands of Red-crowned Cranes occurred in the lower part of the RSZ and corresponded to the upper portion of their low-altitude daily flight-height distribution. Previous studies have shown that large waders usually fly in higher airspace during long distance migration (Galtbalt et al., 2025), with relatively little overlap with the RSZ. In contrast, bird flight during the non-migratory season is more characterized by frequent low-altitude, short distance commutes between foraging sites and habitats (Cleasby et al., 2015; McDuie et al., 2019), so it is more likely to have a high degree of overlap with the RSZ. At the same time, coastal wetlands are flat and open, with almost no vertical obstacles. This environmental setting may further increase the use of the lower height range of the RSZ by Red-crowned Cranes (Aurbach et al., 2018; Skov et al., 2025). Overall, potential collision risk at the local scale depends not only on the structure of the wind turbines, but is also strongly influenced by species-specific patterns of flight height use during particular behavioral stages.

### Implications for wind-energy planning

Identifying key risk height bands is important for wind farm planning and turbine-design. This study shows that increasing hub height can shift the entire RSZ upward, substantially reducing its vertical overlap with the low-altitude flight range of Red-crowned Cranes and thereby lowering the overall potential collision risk. This suggests that the key to turbine-design is not only to reduce the total amount of risk space, but more importantly to change the relative position between the RSZ and the height bands most frequently used by the species of concern. At the local scale, ecological optimization of wind farms should consider not only the horizontal proximity between wind turbines and key bird activity areas (Schwemmer et al., 2023; Hu et al., 2025), but also the flight height of key species and the distribution of important habitats (Diffendorfer et al., 2019; Pearse et al., 2021). At a broader scale, turbine layouts with higher capacity and lower density may also reduce risk exposure per unit area, but their effect still depends on how the target species uses flight height and where the key risk height bands are located within the RSZ (Huso et al., 2021). However, raising hub height should not be regarded as a universally preferable solution. Taller towers may increase construction and maintenance requirements and associated life-cycle costs (Canet, Guilloré & Bottasso, 2023), while their greater visual prominence may affect landscape amenity and acceptance among nearby communities (Guo, Wenz & Auffhammer, 2024). Therefore, decisions to increase hub height should balance potential ecological benefits against engineering feasibility, economic costs, landscape effects, and local community concerns. Structural adjustments could also be complemented by seasonal or time-of-day curtailment, including camera- or radar-triggered shutdowns when cranes approach turbines; selective or automated curtailment has reduced collision mortality in soaring birds at some wind farms (McClure et al., 2021; Ferrer et al., 2022). Increasing blade visibility may provide an additional option (May et al., 2020), although the effectiveness and costs of these measures require species- and site-specific evaluation.

## Limitations

Although satellite tracking provides an important basis for quantifying bird flight height, this study still has several limitations. Previous studies have shown that factors such as wind speed, visibility, and topography may affect bird flight height and collision risk (Willmott, Forcey & Hooton, 2015; Santos et al., 2022; Kamata et al., 2023), but these factors are difficult to fully include in the analysis at the current spatial and temporal resolution of the data. Future studies could further integrate weather conditions and behavioral state information to build a more detailed collision risk assessment framework and test whether key risk height bands are consistent across different species and ecosystems.

## Conclusions

This study shows that the potential collision risk between Red-crowned Cranes and wind turbines in coastal wetlands is mainly concentrated in the 25–50 m height band in the lower part of the turbines, and that increasing hub-height by 20 m can reduce the risk by 48.9%. The formation of collision risk mainly depends on the coupling between bird flight height use and the vertical structure of the turbine rotor-swept zone. Future ecological optimization of wind power should focus on the overlap between the flight heights of key species and the key risk height bands of wind turbines, and develop targeted turbine-design and mitigation strategies accordingly.

##  Supplemental Information

10.7717/peerj.21762/supp-1 Data S1 Anonymized Red-crowned Crane flight-height data Anonymized flight-height dataset used for the vertical collision risk analysis. Geographic coordinates were removed to protect the endangered Red-crowned Crane, and the dataset includes timestamps and flight heights above ground level.

10.7717/peerj.21762/supp-2 Supplemental Information 2 R code for analysis R script used for kernel density estimation, rotor-swept-zone risk calculations, and simulations of alternative turbine hub heights.

10.7717/peerj.21762/supp-3 Table S1 Collision risk across rotor-swept-zone height bands Relative flight-height use, mean rotor-swept chord length, and risk contribution for all height bands within the RSZ.

## Additional Information and Declarations

### Data Availability

The following information was supplied regarding data availability: The data is available in the Supplemental Files.
